# Comparative genomic analysis of Latilactobacillus sakei strains provides new insights into their association with different niche adaptations

**DOI:** 10.1099/mic.0.001578

**Published:** 2025-07-03

**Authors:** Kohei Ito, Yutaro Ito

**Affiliations:** 1 BIOTA Inc., Tokyo, 101-0022, Japan

**Keywords:** codon usage bias, CRISPR-Cas, fermentation, *Latilactobacillus sakei*, plasmids

## Abstract

*Latilactobacillus sakei*, a lactic acid bacterium in diverse environments such as fermented foods, meat and the human gastrointestinal tract, exhibits significant genetic diversity and niche-specific adaptations. This study conducts a comprehensive comparative genomic analysis of 29 complete *L. sakei* genomes to uncover the genetic mechanisms underlying these adaptations. Phylogenetic analysis divided the species into three distinct clades that did not correlate with the source of isolation and did not suggest any niche-specific evolutionary direction. The pan-genome analysis revealed a substantial core genome alongside a diverse genetic repertoire, indicating both high genetic conservation and adaptability. Predicted growth rates based on codon use bias analysis suggest that *L. sakei* strains have an overall faster growth rate and may be able to efficiently dominate in competitive environments. Plasmid analysis revealed a variety of plasmids carrying genes essential for carbohydrate metabolism, enhancing *L. sakei*’s ability to thrive in various fermentation substrates. It was also found that the number of genes belonging to the GH1 family amongst sugar metabolism-related genes present on chromosomes and plasmids varies between strains and that AA1, which is involved in alcohol oxidation, has been acquired from plasmids. blast analysis revealed that some strains have environmental adaptation gene clusters of cell surface polysaccharides that may mediate attachment to food and mucosa. The knowledge gleaned from this study lays a solid foundation for future research aimed at harnessing the genetic traits of *L. sakei* strains for industrial and health-related applications.

## Introduction

*Latilactobacillus sakei* has been isolated from various environments including fermented foods. Specifically, *L. sakei* has been found in meat [1], Japanese *sake* [2,3] and fermented vegetables [4]. It has also been found in human faeces [5]. * L. sakei* produces lactic acid, which acidifies food products and inhibits undesirable autochthonous microbes, allowing it to dominate in a variety of fermented foods [6]. Consequently, certain strains of *L. sakei* are used as starter cultures for the fermentation process [7,8].

*L. sakei* possesses several beneficial functions, including the production of bacteriocins that inhibit food-borne pathogenic bacteria such as *Listeria monocytogenes*, *Enterococcus faecalis* and *Staphylococcus aureus* [9]. Moreover, *L. sakei* is gaining attention for its probiotic functions, including regulation of the gut environment, prevention of inflammation and reduction of obesity [10,11].

Environmental conditions significantly influence the genetic characteristics and evolutionary direction of bacteria, so understanding the phenotype and adaptation mechanisms of *L. sakei* strains is crucial. It is likely that *L. sakei* exhibits different adaptations for each fermented food. Indeed, previous studies have identified three distinct phylogenetic lineages of *L. sakei* using MLST, influenced by their different habitats [12]. Genes that are key for survival in meat products have been conserved in *L. sakei* isolated from these environments [13]. Large-scale comparative genomic analyses using whole genomes are necessary to further investigate the mechanisms of *L. sakei* adaptation to different environmental niches.

Comparative genomic analysis is a crucial method to reveal the genetic diversity and adaptation of *L. sakei* to different niches. Some studies have highlighted the role of codon usage bias in bacterial genomes as an indicator of environmental niche adaptation [14,15]. The previous study [16] has carried out a comparative genomic analysis of *L. sakei* strains but has only focused on the chromosomal genome and has not considered the effect of plasmids on environmental adaptation. In this study, we analysed the pan-genome, phenotype prediction, CRISPR-Cas systems and plasmids of 29 publicly available complete genomes of *L. sakei* from the National Center for Biotechnology Information (NCBI) using bioinformatics approaches.

## Methods

### Genome sequencing, assembly and annotation

For comparative analysis, RefSeq data for all publicly available complete genomes of *L. sakei*, *Carnobacterium* spp., *Hafnia* spp., *Leuconostoc* spp., *Rahnella* spp. and *Serratia* spp. used for comparison of codon bias analysis were downloaded from the NCBI FTP site (https://www.ncbi.nlm.nih.gov/, accessed on 30 April 2023). In order to identify genomic features or plasmid detections with high accuracy, only complete genomes were used for comparative analysis in this study. The final data set consisted of all *L. sakei* strains as shown in Table 1. Protein-coding DNA sequences (CDSs) were predicted, and functional annotations (gene and product names) were assigned using Bakta (v.1.7.0) [17]. CRISPR arrays were detected using CRISPRCasFinder (version 4.2.30) [18] with the default parameters. CRISPR arrays with high evidence levels were considered as highly likely candidates, so we restricted the analysis to CRISPRs with evidence level ≥4 and containing at least one Cas protein.

**Table 1.** Genomic information of *L. sakei* strains

Strain	Size (Mb)	GC%	Assembly	Source	Detail
23K	1.88	41.30	GCA_000026065.1	Meats	
CBA3614	2.02	41.13	GCA_009676365.1	Fermented foods	Kimchi
CBA3635	2.06	41.10	GCA_014081765.1	Fermented foods	Vegetables
CNSC001WB	2.09	40.97	GCA_029677405.1	Fermented foods	Kimchi
FAM18311	2.06	41.01	GCA_002224565.1	Meats	
FLEC01	1.96	41.24	GCA_900234345.1	Human faeces	
J54	2.01	41.31	GCA_900234395.1	Meats	
J64	2.10	41.06	GCA_900234355.1	Meats	
LK-145	1.99	41.15	GCA_002370375.1	Fermented foods	Japanese sake
LT-13	1.94	41.18	GCA_002370355.1	Fermented foods	Japanese sake
LZ217	2.02	41.22	GCA_003627875.1	Fermented foods	Vegetables
MBEL1397	1.99	41.00	GCA_010092945.1	Fermented foods	Kimchi
MFPB16A1401	2.04	41.09	GCA_900234375.1	Meats	
MFPB19	2.06	41.05	GCA_900234405.1	Meats	
ob4.1	2.03	41.10	GCA_018437525.1	Human faeces	
TMW1.114	1.98	41.21	GCA_023734315.1	Fermented foods	Starter culture
TMW1.1189	1.94	41.20	GCA_023734235.1	Fermented foods	Unknown
TMW1.1239	1.98	41.20	GCA_023734255.1	Fermented foods	Bread
TMW1.1396	1.94	41.27	GCA_023734275.1	Fermented foods	Starter culture
TMW1.1398	2.07	41.13	GCA_023734295.1	Fermented foods	Starter culture
TMW1.417	1.98	41.21	GCA_023734335.1	Fermented foods	Starter culture
TMW1.46	2.08	41.08	GCA_023734195.1	Fermented foods	Starter culture
TMW1.578	1.98	41.21	GCA_023734355.1	Fermented foods	Starter culture
WiKim0063	2.08	41.17	GCA_002250035.1	Fermented foods	Vegetables
WiKim0072	2.03	41.17	GCA_003288195.1	Fermented foods	Kimchi
WiKim0074	2.04	41.03	GCA_003288235.1	Fermented foods	Kimchi
WiKim0095	2.12	41.01	GCA_024022895.1	Fermented foods	Kimchi
ZFM225	2.02	41.22	GCA_003627235.1	Fermented foods	Milk
ZFM229	2.02	41.22	GCA_003627315.1	Fermented foods	Vegetables

### Codon usage analysis

To correlate strains with general lifestyle adaptation, we used the R package gRodon2 (version 2.3.0) [19] which uses codon usage bias to estimate growth rates. From a codon usage bias perspective, gRodon identifies the optimization of highly expressed genes, which serves as a robust indicator of selection for growth rates. The relationship between the minimal doubling times (MDTs) and the codon usage bias of each highly expressed gene relative to all other genes (CUBHE) was assessed using Spearman’s rank correlation coefficient. This non-parametric method was chosen due to the potential non-normal distribution of the data. Spearman’s rank correlation coefficient was calculated using the cor() function in R, with the method argument set to ‘spearman’. The statistical significance of the correlation was evaluated using the cor.test() function in R, which provides both the correlation coefficient and its associated *P*-value. Subsequently, pairwise comparisons between genera were performed using Dunn’s test to determine whether MDTs differed significantly amongst genera. To control for false discovery due to multiple comparisons, the Benjamini–Hochberg correction was applied to the resulting *P*-values. A *P*-value and an adjusted *P*-value<0.05 were considered statistically significant.

### Pan-genome analysis

Pan-genomes were constructed using Panaroo (version 1.3.3) [20] in ‘sensitive’ mode with the initial clustering stage at 98% length and the family sequence identity threshold at 70%. Core genes were defined as genes present in ≥95% of the *L. sakei* strains.

To assess the openness of the pan-genome and the stability of the core genome, we constructed gene accumulation curves using the gene presence/absence matrix using Panstripe R packages using a generalized linear model, which was performed [21]. The order in which genomes were added was randomized 100 times, and the number of gene clusters was recorded at each step for both the pan-genome and core genome.

To model the expansion of the pan-genome, we fitted the data to Heap’s law:


$$n=\kappa \cdot N^{\alpha }$$

where n is the total number of gene clusters, N is the number of genomes and κ and α are constants estimated by non-linear least squares fitting.

For the core genome, we used an exponential decay model of the form:


$$n=a\cdot e^{-bN}+c$$

where a, b and c are fitted parameters. The fitted curves and parameter values (including R2) were displayed directly in the accumulation plot. All analyses were performed using custom scripts and the nls() function in R for model fitting.

### Phylogenetic analysis

To infer their phylogenetic relationships, the identified single-copy orthologues were aligned using MAFFT (v7.526) [22], and a phylogenetic tree was reconstructed using the GTR+CAT model of FastTree v2.1.11 [23]. The reliability of each split in the trees was estimated by computing the local support values with the Shimodaira–Hasegawa test. The phylogenetic tree was plotted using ggtree (version 3.10.1) [24,25]. Functional categorization of genes was performed using annotation against the Cluster of Orthologous Groups of proteins (COGs) database based on blast [26].

### Plasmid identification

MOB-suite (v 3.1.2) [27] was used for the typing and reconstruction of plasmid sequences from *L. sakei* strains. Plasmids are mobile genetic elements that allow bacteria to rapidly evolve and adapt to new niches through the horizontal transfer of novel traits to different genetic backgrounds. The plasmid sequence output from the MOB-suite was gene-annotated in Bakta in the same way as chromosomal genomes.

### CAZymes, bacteriocin and environmental adaptation gene annotation

CAZyme annotation was performed using the dbCAN3 annotation tool based on the HMMER search [28]. In the analysis targeting the whole genome, CAZymes with a total of at least four genes per family across all species were used for the analysis. In the analysis using only plasmids, all annotated CAZymes were used. The identity of annotated genes was obtained with an e-value cutoff of 1e-10 or lower. Bacteriocins were annotated using BAGEL4 with whole-genome sequencing [29]. The cell adherence, alcohol dehydrogenase, acid tolerance and salt tolerance-related genes were annotated using local blast [30,33]. The query sequences were obtained from CR936503, CP003032.1 and RBAI00000000. The heatmap was generated using matplotlib 3.8.4 and seaborn 0.13.2 [34].

## Results

### Genomic features of *L. sakei* strains

To date, all complete genomes of *L. sakei* strains have been isolated from different environments, such as human faeces, fermented food, meat and milk (Table 1). The genome size of 29 *L*. *sakei* strains ranged from 1.88 Mbp (strain 23K) to 2.12 Mbp (strain WiKim0095) with an average size of 2.02 Mb. The average G+C content was 41.14 mol%, ranging from 40.97% (strain CNSC001WB) to 41.31% (strain J54).

Genome annotation using Bakta revealed an average of 1,984 CDSs per strain (Table S1, available in the online Supplementary Material). Amongst these, only an average of 32 were annotated as hypothetical proteins, indicating that ~98% of the predicted genes were assigned a putative function. The number of pseudogenes varied significantly between strains, ranging from none in strain WiKim0063 to as many as 25 in strains TMW1.1189 and TMW1.1398. In contrast, no notable differences were observed in the annotation of other genomic features across strains.

All *L. sakei* strains were analysed using the CRISPR-Cas system, and only strain J54 was identified as CRISPR at the evidence level 4. Strain J54 had a complete CRISPR-Cas system, all of which belonged to class IIA including Cas1, Cas2, Cas9 and Csn2 (Table S2).

**Fig. 1.**
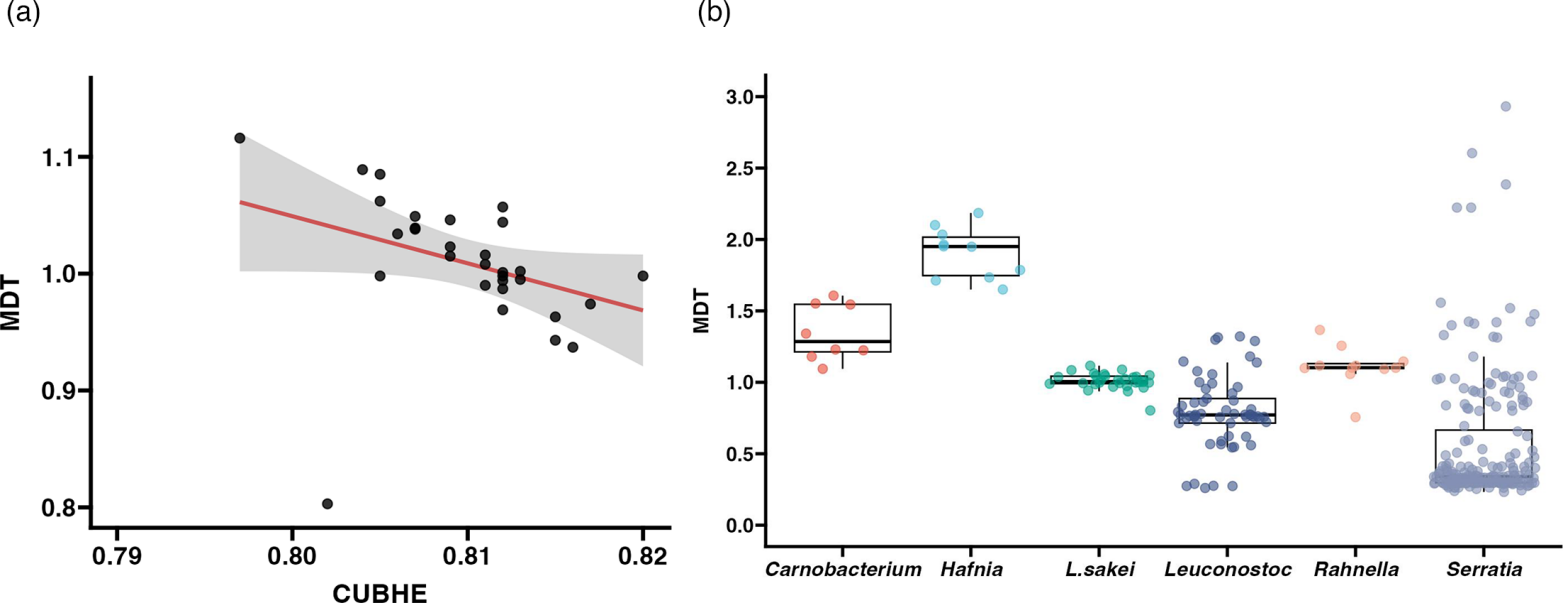
(**a**) Dot plot of MDT and the mean of CUBHE. The red line indicates the linear regression fit, and the grey band shows its 95% CI. (**b**) Boxplot of MDT between some fermented food-associated bacteria.

To investigate more information about the replication abilities of *L. sakei* strains, we performed codon usage bias analysis using gRodon2, a CUB-based tool, to calculate MDT in hours (Fig. 1a). The mean of the CUBHE of *L. sakei* strains ranged from 0.797 (strain LK-145) to 0.817 (strain J54) with an average CUBHE of 0.809. MDT of *L. sakei* strains ranged from 0.803 (strain FLE01) to 1.116 (strain LK-145) with an average MDT of 1.009. CUBHE and MDT were significantly negatively correlated (Spearman’s ρ = −0.603, *P*<0.05), suggesting that higher codon usage bias in highly expressed genes is associated with faster predicted growth. To contextualize the growth potential of *L. sakei*, we compared its predicted MDTs with those of other bacterial genera commonly found in fermented foods in a previous study [35] (Fig. 1b). The MDTs of *L. sakei* were significantly lower than those of *Leuconostoc* (adjusted *P*=0.011) and *Serratia* (adjusted *P*<0.001), but not significantly different from *Carnobacterium* (adjusted *P*=0.266), *Hafnia* (adjusted *P*=0.065) or *Rahnella* (adjusted *P*=0.539). These findings indicate that whilst *L. sakei* does not exhibit the fastest doubling times amongst all tested genera, its growth potential is significantly greater than that of several environmentally and fermentation-associated taxa, particularly *Leuconostoc* and *Serratia*.

### Pan-genome analysis

**Fig. 2.**
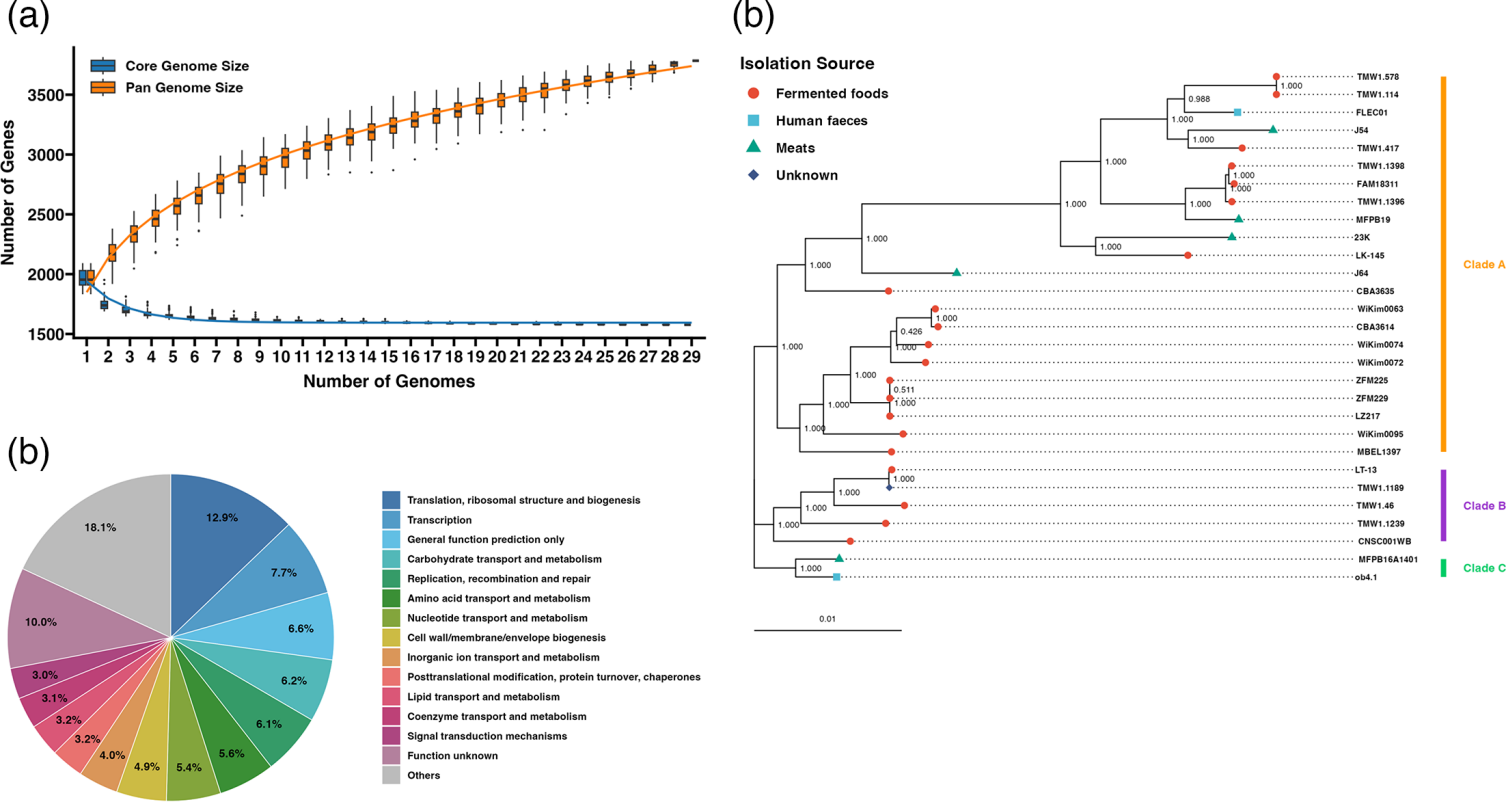
Pan-genome analysis of *L. sakei* strains. (**a**) Accumulation curves of the pan-genome and core genome across increasing numbers of genomes. (**b**) Pie chart based on the database of COGs. (**c**) Phylogenetic tree based on the core gene set. Branch support values shown at each node represent Shimodaira-Hasegawa-like local support values. Clades A, B and C are shown in green, blue and yellow, respectively.

To investigate the genetic diversity amongst *L. sakei* strains, the number of core genes and pan-genomes and the number of strains were plotted (Fig. 2a). It was shown that as the number of *L. sakei* strains increased, the number of pan-genes increased continuously, and the number of core genes tended to be stable. When all strains were added, the total number of genes was stable at 3,784, and the number of core genes reached 1,577. Since the results of the pan-genome analysis are heavily influenced by annotation errors, we considered whether there is evidence for a temporal signal in the gene gain and loss pattern. Specifically, after fitting a panstripe model to the pan-genome, we assessed the significance of the temporal signal by calculating *P*-values of the association between core branch length and gene increase/decrease. The *P*-value indicates that there is a significant association between core branch lengths and gene gain/loss (*P*-value<0.005). These results show an asymptotic trend indicating that *L. sakei* has an open pan-genome.

Functional analysis of the core genes of *L. sakei* strains revealed that their core genome contains more than 5.0% of ‘Translation, ribosomal structure and biogenesis’, ‘Transcription’, ‘Carbohydrate transport and metabolism’, ‘Replication, recombination and repair’ and ‘Amino acid transport and metabolism’. Amongst them, genes related to ‘Carbohydrate transport and metabolism’ accounted for 6.2% of core genes, and 5.6% of core genes were related to ‘Amino acid transport and metabolism’; however, 10.0% of core gene functions are ‘Function unknown’, and 18.1% of core gene functions are ‘Others’ (Fig. 2b).

The phylogenetic tree was constructed based on the core gene set to explore the phylogenetic relationships of all *L. sakei* strains (Fig. 2c). The phylogenetic tree was not divided into different clades for each source of bacterial isolates. In clade A, strains were from fermented foods, meats and human faeces. Clade B was isolated from fermented foods, whilst clade C was isolated from meats and human faeces. *L. sakei* from human faeces (strains FLEC01 and ob4.1) were separated into two different clades.

### Detection of plasmids and their functions in *L. sakei* strains

Amongst all *L. sakei* strains, 39 plasmid sequences were detected in 23 strains (Table S3). In fact, eight strains had no plasmids detected. The G+C content of sequenced plasmids is around 34 or 44 mol%, and the genome size of the plasmids ranged from 1,526 to 93,254 bp as shown in Table S3. Strains FLEC01, LK-145 and MFPB19 have three plasmids, and the replication types of the multiple plasmids in the same bacterial strain were all different. Strain WiKim0095 isolated from kimchi has a plasmid associated with *Latilactobacillus curvatus*. Strain CNSC001WB isolated from watery radish kimchi has a plasmid associated with *Lactiplantibacillus plantarum*.

A list of gene annotations found in more than ten plasmids was generated (Table S4). Ten plasmids have some metabolic pathways. Beta-phosphoglucomutase (beta-PGM) and maltose phosphorylase (MP) are involved in starch and sucrose metabolism. Aldose 1-epimerase is involved in the glycolytic system and glycogenesis.

### Genotype/phenotype association analysis to estimate adaptation to different environments

**Fig. 3.**
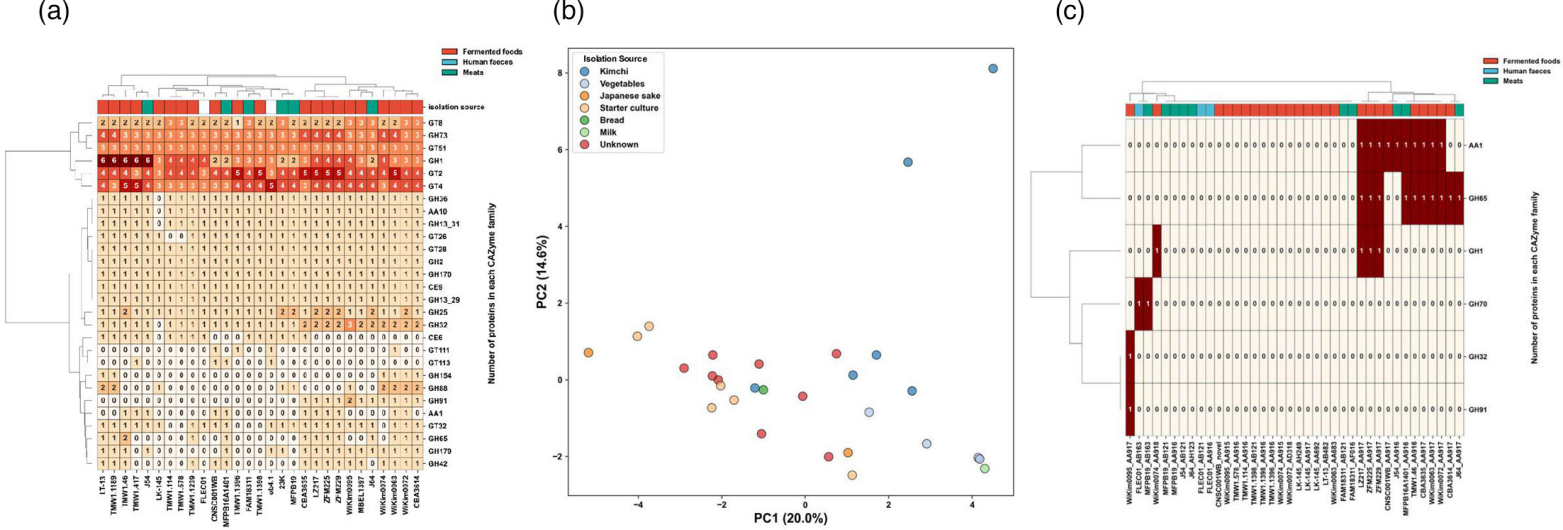
Heatmap of CAZymes. (**a**) Heatmap of CAZymes based on the whole genomes of *L. sakei* strains from each isolation source. The whole genomes of *L. sakei* strains from each isolation source. (**b**) PCA of CAZyme profiles for *L. sakei* strains isolated from different fermented food sources. (**c**) Heatmap of CAZymes constructed from the plasmids of *L. sakei* strains isolated from various sources. The plasmids of *L. sakei* strains from each isolation source.

CAZymes play a role in the synthesis of sugar complexes, oligosaccharides and polysaccharides, as well as the decomposition of complex carbohydrates. Some strains derived from fermented foods and from meat were each clustered into their own clade based on the number of genes in the CAZy family (Fig. 3a). In strains isolated from fermented foods, Principal Component Analysis (PCA) suggested that strains originating from the same source, such as starter culture or vegetables, exhibited similar CAZyme profiles (Fig. 3b). Strains derived from kimchi also tended to form a cluster, but the plot distances between the strains were relatively large. GT8, GH73, GT51, GH1, GT2 and GT4 were the main CAZy families in the whole genome of *L. sakei*. Some CAZymes, such as AA1, GH65, GH1, GH70, GH32 and GH91, existed on the plasmid (Fig. 3c).

**Fig. 4.**
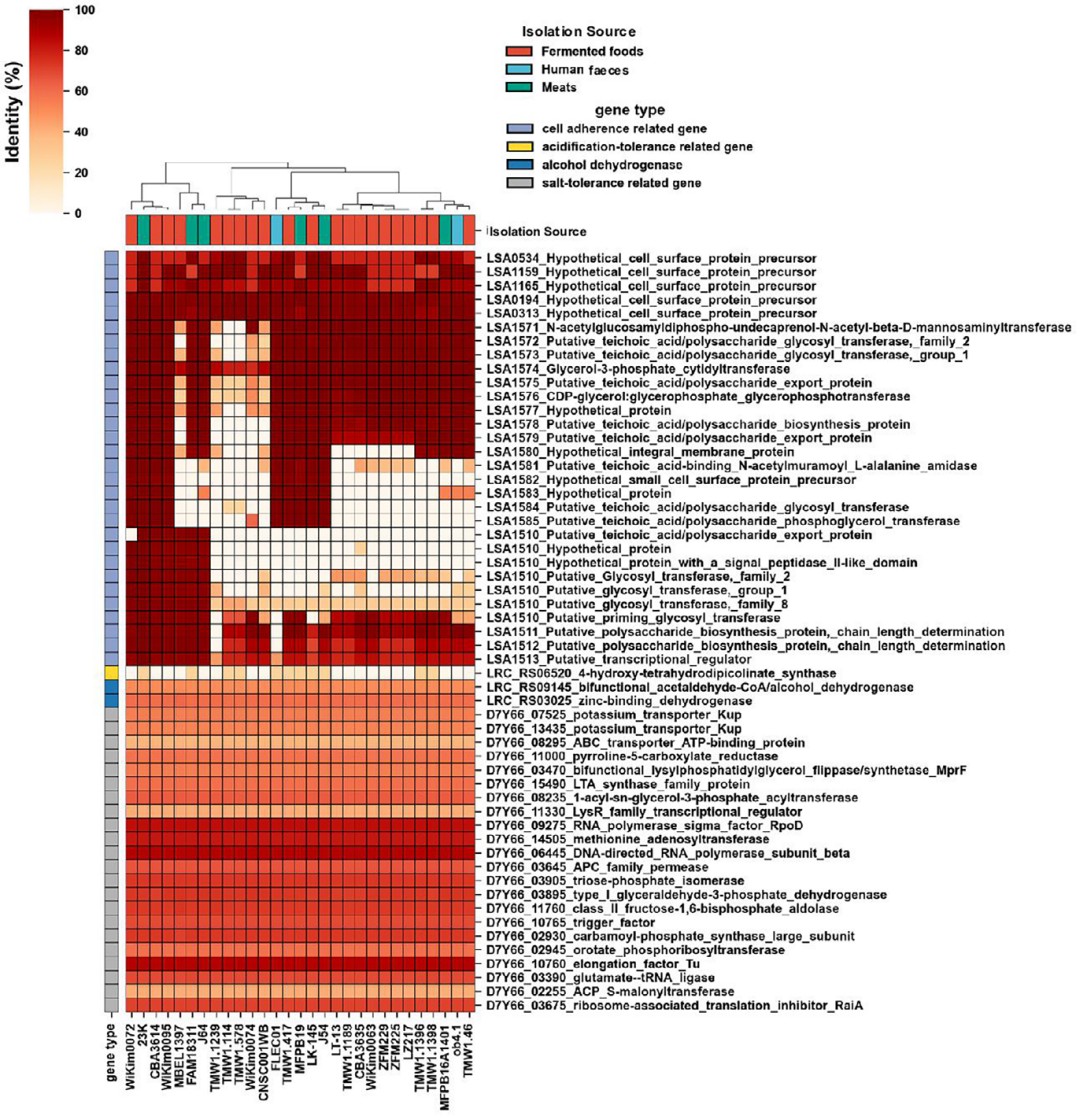
Heatmap of the environmental adaptation genes in the whole genome of *L. sakei* strains from various isolation sources.

In addition, the distribution of genes related to environmental adaptation (cell adhesion, alcohol dehydrogenase, acid tolerance and salt tolerance-related genes) revealed that these gene groups are also highly conserved regardless of the source of isolation (Fig. 4). Some cell adhesion-related gene clusters, potentially involved in the production of surface polysaccharides (LSA1571 to LSA1585 and LSA1510 to LSA1513), vary in presence amongst different strains. Eleven strains such as TMW1.114 and FAM18311 suggested the potential presence of an acid-tolerance gene, namely, *4-hydroxy-tetrahydrodipicolinate synthase*, which regulates lysine metabolism, despite their low sequence identity. On the other hand, it was suggested that salt tolerance and alcohol dehydrogenase-related genes are present in all strains. Although there were no specific characteristics based on the isolation source, several bacteriocins were detected in each strain (Table 2). The bacteriocin detected in the most strains is Carnoicin CP52, found in LT-13, TMW 1.46, MFPB 16A1401 and TMW 1.1189. Sakacin P was detected in TMW1.114, TMW1.578 and J54. Sakacin G was possessed only by FLEC01. FAM18311 exclusively possessed lactocin S and thiopeptide. These bacteriocins were found exclusively on the chromosome, not on the plasmid.

**Table 2.** Bacteriocin in *L. sakei* strains

	Bacteriocin				
	Carnocin CP52	Sakacin P	Sakacin G	Lactocin S	Thiopeptide
**Fermentation foods**					
CBA3614	−	−	−	−	−
CBA3635	−	−	−	−	−
CNSC001WB	−	−	−	−	−
FAM18311	−	−	−	+	+
LK-145	−	−	−	−	−
LT-13	+	−	−	−	−
LZ217	−	−	−	−	−
MBEL1397	−	−	−	−	−
TMW1.114	−	+	−	−	−
TMW1.1239	−	−	−	−	−
TMW1.1396	−	−	−	−	−
TMW1.1398	−	−	−	−	−
TMW1.417	−	−	−	−	−
TMW1.46	+	−	−	−	−
TMW1.578	−	+	−	−	−
WiKim0063	−	−	−	−	−
WiKim0072	−	−	−	−	−
WiKim0074	−	−	−	−	−
WiKim0095	−	−	−	−	−
ZFM225	−	−	−	−	−
ZFM229	−	−	−	−	−
TMW1.1189	+	−	−	−	−
**Meats**					
23K	−	−	−	−	−
J54	−	+	−	−	−
J64	−	−	−	−	−
MFPB16A1401	+	−	−	−	−
MFPB19	−	−	−	−	−
**Human faeces**					
FLEC01	−	−	+	−	−
ob4.1	−	−	−	−	−

## Discussion

The comprehensive genomic analysis of *L. sakei* strains has provided significant insights into their genetic diversity, evolutionary relationships and adaptations to various environments. The analysis of 29 complete genomes isolated from diverse environments, including fermented food, meat and human faeces, highlights the complex and adaptive nature of *L. sakei* strains.

The variation in genome size and G+C content of *L. sakei* strains was small and consistent with previous studies [16]. Codon usage bias analysis provided insight into the replicative ability and metabolic efficiency of *L. sakei* strains (Fig. 1a). The MDTs of *L. sakei* strains ranged from 0.803 to 1.116 h, indicating a relatively fast predicted growth rate. These values are comparable to those of bacteria detected in the final stage of the *Kimoto*-style fermentation starter [35], suggesting that *L. sakei* has growth characteristics well-suited for its role in fermentation processes (Fig. 1b). The codon usage bias of highly expressed genes (CUBHE) in *L. sakei* was negatively correlated with MDT, indicating that strains with more optimized codon usage tend to grow more rapidly. This supports the idea that *L. sakei* has evolved translational features that promote efficient growth under nutrient-rich or competitive conditions. In addition, statistical comparisons revealed that *L. sakei* strains had significantly shorter MDTs than other fermentation-associated genera such as *Hafnia* and *Carnobacterium*, further supporting their ecological advantage during the early stages of microbial succession. This rapid growth potential is particularly important in the context of microbial competition. Several studies have shown that adventitious microbes – those unintentionally introduced from the environment – can colonize fermentation ecosystems [35,38]. In such contexts, fast-growing lactic acid bacteria like *L. sakei* may gain a foothold by quickly dominating the environment and creating acidic conditions that inhibit the growth of these undesirable competitors.

CRISPR-Cas systems are critical for prokaryotic adaptive immunity, countering infections by DNA and RNA viruses and foreign genes such as plasmids [39,43]. Comparative sequence analysis of *L. sakei* has identified 18 CRISPR genotypes in a previous study [44]. In this study, CRISPR-Cas systems were detected in 14 *L. sakei* strains, with only strain J54 possessing a complete system. Active CRISPR-Cas systems are constantly adding new spacer sequences, so the number of these sequences indicates the level of activity of the system [45]. The number of spacer sequences suggests that the IIC subtype of *L. sakei* is more active and better able to resist the insertion of foreign genes. The J54 strain, which was actually the type II-A subtype, had two plasmids (Tables 2 and S2). The presence of class IIA and IIC CRISPR-Cas systems suggests robust mechanisms to resist foreign genetic elements, although the absence of complete systems in most strains may confer adaptability in dynamic environments such as fermented foods.

The pan-genome analysis revealed a substantial core genome of 1,536 genes, indicating a high level of genetic conservation amongst the strains. The presence of 3,968 total genes underscores the extensive genetic diversity within *L. sakei*, which is likely a result of adaptation to varied environmental conditions. The core genes are primarily involved in essential metabolic processes, such as replication, transcription, translation and various metabolic pathways (Fig. 2b). This core functionality is critical for the survival and dominance of *L. sakei* in different niches. Some studies have reported that the stability and conservation of the core gene set allow bacteria to adapt to various environments [13,46]. These results suggest that *L. sakei* retains redundancy in the basic functions necessary for adaptation to diverse environments. Based on the phylogenetic tree of the core gene set, all *L. sakei* strains were divided into three major clades. There was no evident correlation between each branch and the isolated bacterial source (Fig. 2c), consistent with the results of a previous study [16]. Interestingly, there were no significant differences in the composition of these core gene sets amongst strains isolated from different environments (fermented foods, meat products, human faeces, etc.). This observation suggests that *L. sakei* maintains a common genetic basis that allows it to adapt to a wide range of environments.

Analysis of plasmids in *L. sakei* strains revealed significant variability, with 39 plasmid sequences detected in 22 strains. And plasmids were also detected in strain J54, which harboured the most CRISPR-Cas system genes in this study. Plasmids play an important role in horizontal gene transfer, contributing to the genetic diversity and adaptability of bacteria [47,48]. This plasmid-mediated metabolic versatility likely supports the ability of *L. sakei* to dominate in various fermentation processes and adapt to different substrates.

A list of gene annotations shared by more than ten of all detected plasmids is shown (Table S4). It is reported that beta-PGM is detected in *L. plantarum*. These bacteria possess strong carbohydrate utilization capabilities, facilitated by genes such as beta-PGM, which are crucial for efficient fermentation [49]. The degradation of intracellular maltose in *Lactococcus lactis*, which is found in various lactic acid-fermented foods, is known to occur through the combined efforts of beta-PGM and MP [50]. Aldose 1-epimerase, an enzyme that catalyses the interconversion of the alpha- and beta-terminal isomers of hexose, is reported to be produced by *Petrimonas*, *Prevotella* and *Clostridium* in Chinese strongly flavoured liquor produced by solid fermentation in ground pits [51]. These enzymes are important for maintaining robust fermentation activities and improving product quality.

The presence of genomes and plasmids associated with metabolic pathways, such as starch and sucrose metabolism and glycolysis, indicates their role in enhancing the metabolic capabilities of *L. sakei* (Fig. 3a, b). The glycosyl hydrolases (GHs) and glycosyltransferases (GTs) have potential carbohydrate metabolic activity and play an important role in the flavour of fermented foods [52]. GH1 families including β-glucosidases and β-galactosidases, which play a crucial role in the hydrolysis of glycosidic linkages in various substrates, were found to vary in number amongst different strains of *L. sakei*. The GH1 family is known to be involved in lactose hydrolysis and may be present in many strains for the fermentation of milk-based products [53]. Some strains, such as Wikim0074, LZ217, ZFM225 and ZFM229, also possess one GH1 family gene in plasmid. It was suggested that GH1 might be one of the important CAZymes in fermented foods. PCA revealed that even amongst fermented foods, CAZymes exhibit distinct characteristics depending on the type of single-origin food (Fig. 3b). Strains from kimchi and vegetables, despite being isolated across multiple studies, shared similar CAZymes, suggesting the possibility that strains adapted to fermentation environments specific to the type of food were isolated.

Gene clusters involved in the production of cell surface polysaccharides, which vary amongst strains, may mediate attachment to both food surfaces and mucosal surfaces [31]. The genes (LSA1571 to LSA1585) predicted to be involved in the synthesis of polysaccharide-linked teichoic acid were found in strains isolated from diverse sources, indicating acquisition for various environmental adaptations. The genes (LSA1510 to LSA1513) are potentially involved in transferring polysaccharides to a surface component. These genes are primarily found in strains associated with food-related contexts, such as meat and fermented foods. As a possibility, these clusters are considered to have been acquired for colonization on food surfaces. On the other hand, many of the other adaptive genes are shared across all strains, suggesting a robust species-specific environmental adaptability.

In environmental adaptation, inhibiting the growth of other bacteria is a crucial factor. Bacteriocins are antimicrobial peptides with a proteinaceous nature, exhibiting bactericidal or bacteriostatic activity against closely related species or across genera [54]. The FAM18311 strain had lactocin S and thiopeptide, which exhibit activity against a broad spectrum of Gram-positive bacteria [55,56]. It is possible that they acquired it to suppress the growth of spoilage and foodborne pathogenic bacteria, thereby enhancing their environmental adaptation capabilities. Sakacin P, known as a class IIa bacteriocin produced by several *L. sakei* strains, was also present in the TMD111.4, TMD1.578 and J54 strains in this study. Sakacin P was known to exhibit activity against the food pathogen *L. monocytogenes*, thereby believed to provide an advantage in the food environment by suppressing pathogenic micro-organisms [57]. As previously reported, sakacin P is encoded on plasmids [58], indicating that bacteriocins are often acquired from plasmids as part of environmental adaptation. However, in the strains examined in this study, all bacteriocins were encoded on the chromosome. Based on these results, it was suggested that *L. sakei* can inhibit the growth of bacteria that produce various fermentations in diverse environments, implying its potential for wide-ranging applications in the food industry.

The findings from this study highlight the genetic diversity, adaptive mechanisms, and functional capabilities of *L. sakei* strains. The genomic and plasmid analyses provide valuable insights into how *L. sakei* strains have evolved to thrive in different environments, particularly in fermented foods. Understanding these adaptive traits is essential to optimize the use of *L. sakei* as a starter culture in fermentation processes and to develop strategies to harness its probiotic potential. Future research should focus on exploring the functional implications of the identified genes and plasmids in more detail to fully elucidate the mechanisms underlying the adaptability and probiotic benefits of *L. sakei* strains.

## Supplementary material

10.1099/mic.0.001578 Uncited Supplementary Material 1.

## Abbreviations

beta-PGM: beta-phosphoglucomutase
CAZymes: carbohydrate-active enzymes
COGs: cluster of orthologous groups of proteins
CUBHE: codon usage bias of each highly expressed gene relative to all other genes
GHs: glycosyl hydrolases
GTs: glycosyltransferases
MDTs: minimal doubling times
MP: maltose phosphorylase
NCBI: National Center for Biotechnology Information
PCA: principal component analysis

## References

[1] Hammes WP, Bantleon A, Min S (1990). Lactic acid bacteria in meat fermentation. FEMS Microbiol Lett.

[2] Takahashi M, Morikawa K, Kita Y, Shimoda T, Akao T (2021). Changes in bacterial and chemical components and growth prediction for *Lactobacillus sakei* during Kimoto-style fermentation starter preparation in sake brewing: a comprehensive analysis. Appl Environ Microbiol.

[3] Tsuji A, Kozawa M, Tokuda K, Enomoto T, Koyanagi T (2018). Robust domination of *Lactobacillus sakei* in microbiota during traditional Japanese sake starter yamahai-moto fermentation and the accompanying changes in metabolites. Curr Microbiol.

[4] Zhang S, Zhang Y, Wu L, Zhang L, Wang S (2023). Characterization of microbiota of naturally fermented sauerkraut by high-throughput sequencing. Food Sci Biotechnol.

[5] Dal Bello F, Walter J, Hammes WP, Hertel C (2003). Increased complexity of the species composition of lactic acid bacteria in human feces revealed by alternative incubation condition. Microb Ecol.

[6] Champomier-Vergès M-C, Chaillou S, Cornet M, Zagorec M (2002). Erratum to “*Lactobacillus sakei*: recent developments and future prospects”. Res Microbiol.

[7] Hammes WP, Hertel C (1998). New developments in meat starter cultures. Meat Science.

[8] Mani-López E, Ramírez-Corona N, López-Malo A (2024). *Latilactobacillus sakei* as a starter culture to ferment pepper fruits. Food Human.

[9] Martín I, Barbosa J, Pereira SIA, Rodríguez A, Córdoba JJ (2023). Study of lactic acid bacteria isolated from traditional ripened foods and partial characterization of their bacteriocins. *LWT*.

[10] Lim S, Moon JH, Shin CM, Jeong D, Kim B (2020). Effect of *Lactobacillus sakei*, a probiotic derived from kimchi, on body fat in Koreans with obesity: a randomized controlled study. Endocrinol Metab.

[11] Tatsinkou LLT, Fossi BT, Sotoing GT, Mambou HMAY, Ivo PEA (2023). Prophylactic effects of probiotic bacterium *Latilactobacillus sakei* on haematological parameters and cytokine profile of mice infected with *Plasmodium berghei* ANKA during early malaria infection. Life Sci.

[12] Chaillou S, Lucquin I, Najjari A, Zagorec M, Champomier-Vergès M-C (2013). Population genetics of *Lactobacillus sakei* reveals three lineages with distinct evolutionary histories. PLoS One.

[13] Nyquist OL, McLeod A, Brede DA, Snipen L, Aakra Å (2011). Comparative genomics of *Lactobacillus sakei* with emphasis on strains from meat. Mol Genet Genomics.

[14] Botzman M, Margalit H (2011). Variation in global codon usage bias among prokaryotic organisms is associated with their lifestyles. Genome Biol.

[15] Willenbrock H, Friis C, Juncker AS, Ussery DW (2006). An environmental signature for 323 microbial genomes based on codon adaptation indices. Genome Biol.

[16] Chen Y, Li N, Zhao S, Zhang C, Qiao N (2021). Integrated phenotypic-genotypic analysis of from different niches. Foods.

[17] Schwengers O, Jelonek L, Dieckmann MA, Beyvers S, Blom J (2021). Bakta: rapid and standardized annotation of bacterial genomes via alignment-free sequence identification. Microb Genom.

[18] Couvin D, Bernheim A, Toffano-Nioche C, Touchon M, Michalik J (2018). CRISPRCasFinder, an update of CRISRFinder, includes a portable version, enhanced performance and integrates search for Cas proteins. Nucleic Acids Res.

[19] Weissman JL, Hou S, Fuhrman JA (2021). Estimating maximal microbial growth rates from cultures, metagenomes, and single cells via codon usage patterns. Proc Natl Acad Sci USA.

[20] Tonkin-Hill G, MacAlasdair N, Ruis C, Weimann A, Horesh G (2020). Producing polished prokaryotic pangenomes with the Panaroo pipeline. Genome Biol.

[21] Tonkin-Hill G, Gladstone RA, Pöntinen AK, Arredondo-Alonso S, Bentley SD (2023). Robust analysis of prokaryotic pangenome gene gain and loss rates with Panstripe. Genome Res.

[22] Katoh K, Standley DM (2013). MAFFT multiple sequence alignment software version 7: improvements in performance and usability. Mol Biol Evol.

[23] Price MN, Dehal PS, Arkin AP (2010). FastTree 2--approximately maximum-likelihood trees for large alignments. PLoS One.

[24] Xu S, Li L, Luo X, Chen M, Tang W (2022). *Ggtree*: a serialized data object for visualization of a phylogenetic tree and annotation data. *iMeta*.

[25] Yu G, Smith DK, Zhu H, Guan Y, Lam T-Y (2017). Ggtree: an R package for visualization and annotation of phylogenetic trees with their covariates and other associated data. Methods Ecol Evol.

[26] Galperin MY, Makarova KS, Wolf YI, Koonin EV (2015). Expanded microbial genome coverage and improved protein family annotation in the COG database. Nucleic Acids Res.

[27] Robertson J, Nash JHE (2018). MOB-suite: software tools for clustering, reconstruction and typing of plasmids from draft assemblies. Microb Genom.

[28] Zheng J, Ge Q, Yan Y, Zhang X, Huang L (2023). dbCAN3: automated carbohydrate-active enzyme and substrate annotation. Nucleic Acids Res.

[29] van Heel AJ, de Jong A, Song C, Viel JH, Kok J (2018). BAGEL4: a user-friendly web server to thoroughly mine RiPPs and bacteriocins. Nucleic Acids Res.

[30] Altschul SF, Gish W, Miller W, Myers EW, Lipman DJ (1990). Basic local alignment search tool. J Mol Biol.

[31] Chaillou S, Champomier-Vergès M-C, Cornet M, Crutz-Le Coq A-M, Dudez A-M (2005). The complete genome sequence of the meat-borne lactic acid bacterium *Lactobacillus sakei* 23K. Nat Biotechnol.

[32] Pang X, Li W, Yang L, Hu C, Lu J (2019). Whole-Genome Sequencing and Genomic-Based Acid Tolerance Mechanisms of Lactobacillus Delbrueckii Subsp. Bulgaricus LJJ.

[33] Yao W, Yang L, Shao Z, Xie L, Chen L (2020). Identification of salt tolerance-related genes of *Lactobacillus plantarum* D31 and T9 strains by genomic analysis. Ann Microbiol.

[34] Hunter JD (2007). Matplotlib: a 2D graphics environment. Comput Sci Eng.

[35] Ito K, Niwa R, Yamagishi Y, Kobayashi K, Tsuchida Y (2023). A unique case in which Kimoto-style fermentation was completed with *Leuconostoc* as the dominant genus without transitioning to *Lactobacillus*. J Biosci Bioeng.

[36] Ito K, Niwa R, Kobayashi K, Nakagawa T, Hoshino G (2023). A dark matter in sake brewing: origin of microbes producing a Kimoto-style fermentation starter. Front Microbiol.

[37] Kanamoto E, Terashima K, Shiraki Y, Nishida H (2021). Diversity of *Bacillus* isolates from the sake brewing process at a sake brewery. *Microorganisms*.

[38] Terasaki M, Kimura Y, Yamada M, Nishida H (2021). Genomic information of *Kocuria* isolates from sake brewing process. *AIMS Microbiol*.

[39] Barrangou R, Fremaux C, Deveau H, Richards M, Boyaval P (2007). CRISPR provides acquired resistance against viruses in prokaryotes. Science.

[40] Hatoum-Aslan A, Maniv I, Samai P, Marraffini LA (2014). Genetic characterization of antiplasmid immunity through a type III-A CRISPR-Cas system. J Bacteriol.

[41] Jiang W, Samai P, Marraffini LA (2016). Degradation of phage transcripts by CRISPR-associated RNases enables type III CRISPR-Cas immunity. Cell.

[42] Semenova E, Jore MM, Datsenko KA, Semenova A, Westra ER (2011). Interference by clustered regularly interspaced short palindromic repeat (CRISPR) RNA is governed by a seed sequence. Proc Natl Acad Sci USA.

[43] Tamulaitis G, Kazlauskiene M, Manakova E, Venclovas Č, Nwokeoji AO (2014). Programmable RNA shredding by the type III-A CRISPR-Cas system of *Streptococcus thermophilus*. Mol Cell.

[44] Schuster JA, Vogel RF, Ehrmann MA (2019). Characterization and distribution of CRISPR-Cas systems in *Lactobacillus sakei*. Arch Microbiol.

[45] Tyson GW, Banfield JF (2008). Rapidly evolving CRISPRs implicated in acquired resistance of microorganisms to viruses. Environ Microbiol.

[46] Lin H, Yu M, Wang X, Zhang X-H (2018). Comparative genomic analysis reveals the evolution and environmental adaptation strategies of vibrios. BMC Genomics.

[47] Billane K, Harrison E, Cameron D, Brockhurst MA (2022). Why do plasmids manipulate the expression of bacterial phenotypes?. Phil Trans R Soc B.

[48] Heuer H, Smalla K (2012). Plasmids foster diversification and adaptation of bacterial populations in soil. FEMS Microbiol Rev.

[49] Cui Y, Wang M, Zheng Y, Miao K, Qu X (2021). The carbohydrate metabolism of *Lactiplantibacillus plantarum*. Int J Mol Sci.

[50] Qian N, Stanley GA, Hahn-Hägerdal B, Rådström P (1994). Purification and characterization of two phosphoglucomutases from *Lactococcus lactis* subsp. lactis and their regulation in maltose- and glucose-utilizing cells. J Bacteriol.

[51] Ren D, Liu S, Zhang S, Qin H, Han X (2022). Multi-Omics Reveals Microbial Roles and Metabolic Functions at the Spatiotemporal Niche in Pit Mud.

[52] Liang T, Jiang T, Liang Z, Zhang N, Dong B (2023). Carbohydrate-active enzyme profiles of *Lactiplantibacillus plantarum* strain 84-3 contribute to flavor formation in fermented dairy and vegetable products. *Food Chem X*.

[53] Plaza-Vinuesa L, Sánchez-Arroyo A, Moreno FJ, de Las Rivas B, Muñoz R (2023). Dual 6Pβ-galactosidase/6Pβ-glucosidase GH1 family for lactose metabolism in the probiotic bacterium *Lactiplantibacillus plantarum* WCFS1. J Agric Food Chem.

[54] Gaspar C, Donders GG, Palmeira-de-Oliveira R, Queiroz JA, Tomaz C (2018). Bacteriocin production of the probiotic *Lactobacillus acidophilus* KS400. AMB Express.

[55] Cintas LM, Casaus P, Fernández MF, Hernández PE (1998). Comparative antimicrobial activity of enterocin L50, pediocin PA-1, nisin A and lactocin S against spoilage and foodborne pathogenic bacteria. Food Microbiol.

[56] Darbandi A, Asadi A, Mahdizade Ari M, Ohadi E, Talebi M (2022). Bacteriocins: properties and potential use as antimicrobials. J Clin Lab Anal.

[57] Møretrø T, Naterstad K, Wang E, Aasen IM, Chaillou S (2005). Sakacin P non-producing *Lactobacillus sakei* strains contain homologues of the sakacin P gene cluster. Res Microbiol.

[58] Vaughan A, Eijsink VGH, Van Sinderen D (2003). Functional characterization of a composite bacteriocin locus from malt isolate *Lactobacillus sakei* 5. Appl Environ Microbiol.

